# BMP Receptor Signaling Is Required for Postnatal Maintenance of Articular Cartilage

**DOI:** 10.1371/journal.pbio.0020355

**Published:** 2004-10-19

**Authors:** Ryan B Rountree, Michael Schoor, Hao Chen, Melissa E Marks, Vincent Harley, Yuji Mishina, David M Kingsley

**Affiliations:** **1**Department of Developmental Biology and Howard Hughes Medical Institute, Stanford University School of MedicineStanford, CaliforniaUnited States of America; **2**Prince Henry's Institute of Medical Research, Monash Medical CentreClayton, VictoriaAustralia; **3**National Institute of Environmental Health Sciences, National Institutes of HealthResearch Triangle Park, North CarolinaUnited States of America

## Abstract

Articular cartilage plays an essential role in health and mobility, but is frequently damaged or lost in millions of people that develop arthritis. The molecular mechanisms that create and maintain this thin layer of cartilage that covers the surface of bones in joint regions are poorly understood, in part because tools to manipulate gene expression specifically in this tissue have not been available. Here we use regulatory information from the mouse *Gdf5* gene (a bone morphogenetic protein [BMP] family member) to develop new mouse lines that can be used to either activate or inactivate genes specifically in developing joints. Expression of Cre recombinase from *Gdf5* bacterial artificial chromosome clones leads to specific activation or inactivation of floxed target genes in developing joints, including early joint interzones, adult articular cartilage, and the joint capsule. We have used this system to test the role of BMP receptor signaling in joint development. Mice with null mutations in *Bmpr1a* are known to die early in embryogenesis with multiple defects. However, combining a floxed *Bmpr1a* allele with the *Gdf5*-*Cre* driver bypasses this embryonic lethality, and leads to birth and postnatal development of mice missing the *Bmpr1a* gene in articular regions. Most joints in the body form normally in the absence of *Bmpr1a* receptor function. However, articular cartilage within the joints gradually wears away in receptor-deficient mice after birth in a process resembling human osteoarthritis. *Gdf5*-*Cre* mice provide a general system that can be used to test the role of genes in articular regions. BMP receptor signaling is required not only for early development and creation of multiple tissues, but also for ongoing maintenance of articular cartilage after birth. Genetic variation in the strength of BMP receptor signaling may be an important risk factor in human osteoarthritis, and treatments that mimic or augment BMP receptor signaling should be investigated as a possible therapeutic strategy for maintaining the health of joint linings.

## Abstract 2

Through genetic manipulation, these authors have reduced signaling by bone morphogenetic factors in joint regions, and created a valuable model for the study of arthritis

## Introduction

Thin layers of articular cartilage line the bones of synovial joints and provide a smooth, wear-resistant structure that reduces friction and absorbs impact forces (Brandt et al. 1998). Loss or damage to articular cartilage is a hallmark of arthritic diseases and is one of the most common reasons that both young and old adults seek medical care. Millions of people are afflicted with arthritis, and it ultimately affects more than half of people over the age of 65 (Badley 1995; Yelin and Callahan 1995). A better understanding of the molecular mechanisms that create and maintain articular cartilage is crucial for discovering the causes of joint disorders and providing useful medical treatments.

Joint formation begins during embryogenesis, when stripes of high cell density called interzones form across developing skeletal precursors (Haines 1947). Programmed cell death occurs within the interzone, and a three-layered interzone forms that has two layers of higher cell density flanking a region of lower cell density. Non-joint precursors of the skeleton typically develop into cartilage, which hypertrophies and is replaced by bone. However, cells within the high-density layers of the interzone are excluded from this process and develop into the permanent layers of articular cartilage found in the mature joint (Mitrovic 1978).

Studies over the last 10 y have begun to elucidate some of the signaling pathways that contribute to the early stages of joint formation. *Wnt14* is expressed in stripes at the sites where joints will form, and it is capable of inducing expression of other joint markers when misexpressed at new locations in the limb (Hartmann and Tabin 2001). Several members of the bone morphogenetic protein (BMP) family of secreted signaling molecules are also expressed in stripes at sites where joints will form, including those encoded by the genes *Gdf5, Gdf6, Gdf7, Bmp2,* and *Bmp4* (Storm and Kingsley 1996; Wolfman et al. 1997; Francis-West et al. 1999; Settle et al. 2003). Of these, *Gdf5* expression is most strikingly limited to regions where joints will develop and is one of the earliest known markers of joint formation. Mutations in either *Gdf5* or the closely related *Gdf6* gene also block formation of joints at specific locations, providing strong evidence that these molecules are essential for the joint formation process (Storm et al. 1994; Settle et al. 2003). However, mutations in *Bmp2* or *Bmp4* cause early embryonic lethality, making it difficult to test their role in joint formation (Winnier et al. 1995; Zhang and Bradley 1996).

Much less is known about how signaling pathways function during the subsequent maturation and maintenance of adult joint structures. Importantly, BMP signaling components are present in adult articular cartilage, suggesting that they may function during the late development or maintenance of this critical structure (Erlacher et al. 1998; Chubinskaya et al. 2000; Muehleman et al. 2002; Bau et al. 2002; Bobacz et al. 2003).

BMPs bind tetrameric complexes of two type I and two type II transmembrane serine-threonine kinase receptors. Upon BMP binding, these complexes transduce a signal by phosphorylating members of the Smad family of transcription factors (Massague 1996). Recent experiments have implicated two different BMP type I receptors in skeletal patterning, BMPR1A and BMPR1B. Both receptors can bind BMP2, BMP4, and GDF5, although GDF5 shows higher affinity for BMPR1B (Koenig et al. 1994; ten Dijke et al. 1994; Yamaji et al. 1994; Nishitoh et al. 1996; Chalaux et al. 1998). Both receptors are also expressed in dynamic patterns during normal development. In limbs, *Bmpr1a* expression becomes restricted to joint interzones, perichondrium, periarticular cartilage, hypertrophic chondrocytes, and interdigital limb mesenchyme. In comparison, *Bmpr1b* expression is seen primarily in condensing precartilaginous mesenchymal cells, regions flanking joint interzones, perichondrium, and periarticular cartilage (Dewulf et al. 1995; Mishina et al. 1995; Zou et al. 1997; Baur et al. 2000). Null mutations in the *Bmpr1b* gene produce viable mice with defects in bone and joint formation that closely resemble those seen in mice missing *Gdf5* (Storm and Kingsley 1996; Baur et al. 2000; Yi et al. 2000). Null mutations in *Bmpr1a* cause early embryonic lethality, with defects in gastrulation similar to those seen in mice with mutations in *Bmp4* (Mishina et al. 1995; Winnier et al. 1995). Recent studies with floxed alleles suggest that *Bmpr1a* is also required for many later developmental events, but its roles in bone and joint formation have not yet been tested (Mishina 2003).

A genetic system for activating or inactivating genes specifically in joint tissues would be particularly useful for further studies of joint formation and maintenance. Here we take advantage of the tissue-specific expression pattern of the *Gdf5* gene to engineer a *Cre*/*loxP* system (Nagy 2000), *Gdf5-Cre,* that can be used to remove or ectopically express genes in joints. Tests with reporter mice show that this system is capable of modifying genes in all of the structures of the mature synovial joint, including the ligaments of the joint capsule, the synovial membrane, and the articular cartilage. *Gdf5-Cre* recombination bypasses the early embryonic lethality of null mutations in *Bmpr1a,* and shows that this receptor is required for early joint formation at some locations and for initiation of programmed cell death in webbing between digits. Interestingly, *Bmpr1a* is also required for postnatal maintenance of articular cartilage throughout most of the skeleton. In *Gdf5-Cre/Bmpr1a^floxP^* mice, articular cartilage initially forms normally, but subsequently loses expression of several key cartilage markers after birth. It ultimately fibrillates and degenerates, resulting in severe osteoarthritis and loss of mobility. These experiments suggest that BMP signaling is required for normal maintenance of postnatal articular cartilage, and that modulation of the BMP signaling pathway may play an important role in joint disease.

## Results

### Genetic System for Testing the Function of Genes in Joint Development

**Figure 1:**
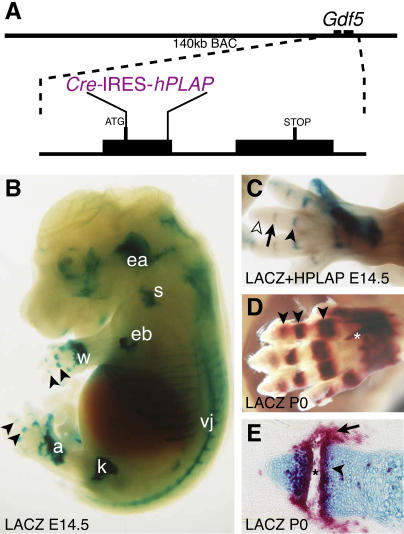
A Genetic System to Drive Gene Recombination in Developing Joints (A) A 140-kb BAC from the *Gdf5* locus was modified by inserting *Cre*-IRES-*hPLAP* into the translation start site of *Gdf5* and used to make transgenic mice. Not to scale. See Materials and Methods for details. (B–E) Visualization of *Gdf5-Cre* driven recombination patterns based on activation of *lacZ* expression from the *R26R* Cre reporter allele. (B) LACZ activity is visible as blue staining in the ear (ea) and the joints of the shoulder (s), elbow (eb), wrist (w), knee (k), ankle (a), vertebra (vj), and phalanges (black arrowheads) of an E14.5 mouse embryo. (C) E14.5 hindlimb double-stained to show both HPLAP expression from the transgene (grey/purple staining) and LACZ expression from the rearranged *R26R* allele (blue staining). Note that both markers are visible in the oldest, proximal interphalangeal joint (black arrowhead), only HPLAP activity is visible in the more recently formed medial interphalangeal joint (black arrow), and neither HPLAP nor LACZ expression is visible in the youngest, most distal joint of the digit (white arrowhead). (D) Newborn (P0) forelimb with skin partially removed showing LACZ activity expressed in all phalangeal joints (red Salmon gal staining, black arrowheads) and regions of some tendons (asterisk). (E) Section through the most distal phalangeal joint of a P0 hindlimb stained with Alcian blue to mark cartilage showing LACZ expression (stained red) in all tissues of developing joints: articular cartilage (black arrowhead), precursors of ligaments and synovial membranes (black arrow), and cells where cavitation is occurring (asterisk).

To generate a general system capable of specifically testing genes for functions in skeletal joint development, we engineered transgenic mice to express Cre recombinase in developing joints (Figure 1). *Gdf5* is a gene strongly expressed in stripes across developing skeletal elements during embryonic joint formation. A bacterial artificial chromosome (BAC) containing the *Gdf5* locus was modified by homologous recombination in bacteria to insert a cassette encoding Cre-internal ribosome entry site (IRES)-human placental alkaline phosphatase (hPLAP) into the translation start site of *Gdf5* (Figure 1A). This modified BAC was then used to make lines of transgenic mice. The resulting *Gdf5-Cre* transgenic mice were tested for transgene expression and Cre recombinase activity by crossing them to R26R reporter mice that activate the expression of *lacZ* after Cre-mediated removal of transcriptional stop sequences (Soriano 1999). The resulting progeny were analyzed both for expression of the transgene by assaying HPLAP activity and for recombination of DNA by assaying LACZ activity. The progeny from all three lines showed strong LACZ expression primarily in joints, and in two of three lines HPLAP expression could also be seen in joint regions. Interestingly, HPLAP expression in the *Gdf5-Cre* transgenic GAC(A) line used for all subsequent breeding experiments was seen to precede LACZ expression during successive development of joints in the digits (Figure 1C) (unpublished data). These experiments clearly demonstrate that the *Gdf5-Cre* transgene expresses Cre recombinase and causes DNA recombination in developing joint regions.

**Figure 2:**
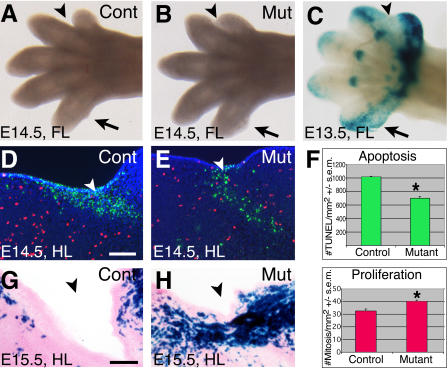
*Bmpr1a* Is Required for Webbing Regression and Apoptosis in Specific Regions of the Limb (A and B) Control E14.5 forelimb (A) compared to a, E14.5 mutant forelimb (B) showing webbing between digits 1 and 2 (arrowheads) and extra tissue at the posterior of digit 5 (arrows). (C) *Gdf5-Cre* induced *lacZ* expression from *R26R* in an E13.5 forelimb showing LACZ staining (blue) in metacarpal-phalangeal joints, between digits 1 and 2 (arrowhead), and in a region posterior to digit 5 (arrow). (D and E) Sections of E14.5 hindlimbs showing apoptosis visualized by TUNEL staining (green) and proliferation visualized by staining for histone H3 phosphorylation (red). Controls show strong, uniform TUNEL staining between digits 1 and 2 (D, arrowhead) while mutants show patchy TUNEL staining interspersed with mitotic cells in similar regions (E). Scale bar = 200 μm. (F) Quantitation of TUNEL staining and mitotic cells in the posterior region of the fifth digit shows apoptosis is reduced 30% while proliferation is increased 20% (asterisks indicate statistically significant difference). (G and H) By E15.5, interdigital tissue has regressed in controls (G, arrowhead). In contrast, tissue remains in mutants at this location, primarily derived from cells that have undergone *Gdf5-Cre*-mediated recombination that inactivates *Bmpr1a* function and activates expression of LACZ (H). Scale bar = 75 μm.

GAC(A) mice were crossed with *lacZ ROSA26 Cre* reporter strain (R26R) mice to analyze the pattern of Cre-mediated *lacZ* recombination throughout development. Joints in developing limbs begin forming in a proximal-distal pattern such that the shoulder joint forms prior to the elbow joint. In addition, three major stages of early joint development have been defined by histology as (1) interzone formation, (2) three-layer interzone formation, and (3) cavitation (Mitrovic 1978). Consistent with the proximal-distal pattern of joint development in the limbs, LACZ activity is seen at embryonic day 12.5 (E12.5) in the more proximal joints, including the shoulder and knee (unpublished data). By E14.5, LACZ expression is typically seen in all but the most distal joints of the limbs (Figure 1B and 1C), but with some variability in both strength and extent of expression from embryo to embryo. The strongest-staining embryos often have additional staining in fingertips (not seen in the E14.5 embryo in Figure 1C, but clearly detectable in the E13.5 embryo shown in Figure 2). Sections through developing joints show that LACZ is present in many cells at the interzone stage (unpublished data). However, expression of LACZ in nearly 100% of joint cells is not achieved until the three-layer interzone stage (for example, in the knee joint at E14.5 or in any of the phalangeal joints at E16.5 (unpublished data). Within the developing skeleton, Cre-mediated expression of LACZ remains strikingly specific to joints throughout development. Furthermore, it is seen in all the structures of postnatal synovial joints including the articular cartilage, joint capsule, and synovial membrane (Figure 1D and 1E) (unpublished data). These patterns are consistent with the well-established expression of *Gdf5* in interzone regions during embryonic development (Storm and Kingsley 1996). Adult expression patterns of the *Gdf5* gene are not as well characterized, but *Gdf5* expression has previously been detected in adult articular cartilage using both RT-PCR and immunocytochemistry (Chang et al. 1994; Erlacher et al. 1998; Bobacz et al. 2002).

Other sites besides limb joints also have Cre-mediated *lacZ* expression. Starting at E13.5, LACZ activity is detected in an anterior and posterior domain of the limb bud (Figure 2C). At E14.5, LACZ activity is detectable in the developing ear pinnae, ribs, sternum, tissues in the face, and some regions of the brain and spinal cord (Figure 1B) (unpublished data). At birth, LACZ is also expressed in tendons running along the vertebral column, regions of tendons in the wrist and ankle, and some tendon insertions (Figure 1D) (unpublished data). By 5 wk of age, LACZ is also expressed in the hair follicles, ear cartilage, some cells in the growth plate of the long bones, and portions of the brain and spinal cord (unpublished data). Surprisingly, 23 of 63, or 37% of transgenic mice analyzed also show some degree of wider “ectopic” LACZ expression, which can extend throughout many different tissues in the animal. However, sustained expression of the transgene itself, as assayed by HPLAP activity, is still restricted primarily to joints in animals that show evidence of more generalized recombination based on LACZ expression (unpublished data). This suggests that in a fraction of animals, sporadic expression of Cre at some time early in development is sufficient to lead to both ectopic recombination and LACZ expression. While the fraction of animals with broader recombination patterns must be tracked and accounted for during experiments, these animals offer the potential benefit of revealing additional new functions of target genes that could be subsequently studied with additional site-specific Cre drivers.

### *Gdf5-Cre/Bmpr1a^floxP^* Animals Survive to Adulthood with Ear, Webbing, and Joint Defects

**Figure 3:**
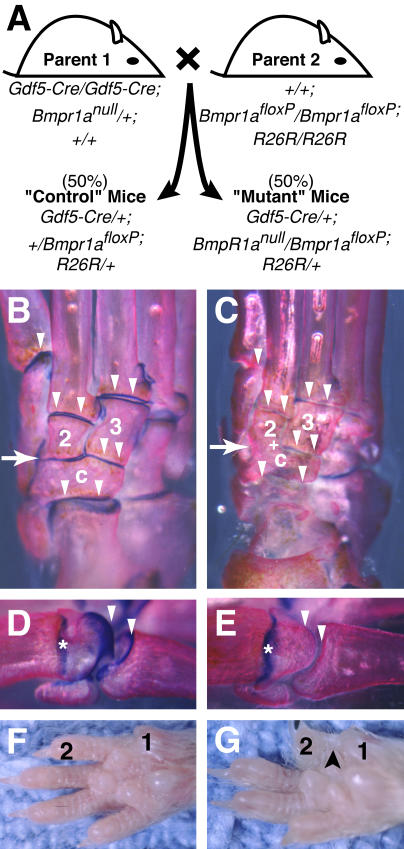
*Gdf5-Cre*-Mediated Deletion of *Bmpr1a* (A) Breeding strategy simultaneously deletes *Bmpr1a^floxP^* and allows visualization of *Gdf5-Cre*-mediated recombination by *lacZ* expression from *R26R*. (B–E) 5-week-old mutant and control mice stained with Alcian blue to mark cartilage and alizarin red to mark bone. (B) Ankle of control with strong blue staining lining each joint (arrowheads). (C) Ankle of mutant showing an absence of blue staining in most regions (arrowheads) and a joint fusion between the central (c) and second (2) tarsals (arrow). (D) Control and (E) mutant metatarsal/phalangeal joint which lacks blue staining in articular regions (arrowheads) but retains staining in the growth plate (asterisks). (F) Control forelimb. (G) Mutant forelimb with webbing between the first and second digit (black arrowhead).

We next used the *Gdf5-Cre* system to test the role of BMP signaling during normal joint development. *Gdf5-Cre* transgenic mice were bred to animals carrying a conditional floxed allele of the *Bmpr1a* locus (Mishina et al. 2002)*,* usually in the presence of the *R26R* reporter allele to facilitate simultaneous visualization of Cre-mediated recombination patterns (see typical cross in Figure 3). PCR amplification confirmed that a key exon of the *Bmpr1a* gene was deleted in mice that also carried the *Gdf5-Cre* transgene (unpublished data). Previous studies have shown that the recombined *Bmpr1a^floxP^* allele mimics a null allele of the *Bmpr1a* locus when transmitted through the germline (Mishina et al. 2002). The *Gdf5-Cre/Bmpr1a^floxP^* conditional knockout mice were viable and survived to adulthood, showing that the *Gdf5-Cre* driver can bypass the early embryonic lethality previously reported in animals with a null mutation in the *Bmpr1a* locus (Mishina et al. 1995).

The viable *Gdf5-Cre/Bmpr1a^floxP^* mice showed several phenotypes. First, the conditional knockout mice had shorter ears that often lay flatter against their heads than controls (controls 13.1 ± 0.1 mm long, *n* = 38; mutants 11.8 ± 0.2 mm, *n* = 11; *p* < 0.0001). BMP signaling is known to be required for growth of the external ear of mice (Kingsley et al. 1992), and this phenotype likely reflects loss of *Bmpr1a* function in the fraction of ear cells that express the *Gdf5-Cre* transgene. Most mutant mice also showed soft tissue syndactyly or retention of webbing between the first and second digits of their feet, a phenotype that was more frequent and more severe in the forelimbs (201 of 220, or 91%, of forefeet and 109 of 220, or 50%, of hindfeet). Finally, mutant animals showed obvious skeletal changes in whole-mount skeletal preparations. At some sites in the ankles, joints seemed to be missing entirely, with fusion of bones that would normally be separate. For example, the second distal tarsal was fused to the central tarsal bone in every conditional knockout animal examined (18 of 18), a phenotype not observed in controls (zero of 18) (Figure 3B and 3C). At other locations, joints had clearly formed but showed dramatic loss of staining with the cartilage matrix marker Alcian blue (Figure 3B–3E) (unpublished data). Normal Alcian blue staining was seen in non-articular regions, such as the cartilaginous growth plate (Figure 3D and 3E, asterisk). These data suggest that *Bmpr1a* function is required for the formation of specific joints in the ankle region and for either generation or maintenance of articular cartilage in most other joints of the limb.

### Developmental Origin of Webbing Phenotype

Interdigital mesenchyme is normally eliminated by apoptosis during embryonic development, a process that can be stimulated by BMP beads, inhibited by Noggin, or blocked by overexpression of dominant-negative BMP receptors (Garcia-Martinez et al. 1993; Yokouchi et al. 1996; Zou and Niswander 1996; Guha et al. 2002). Limbs of *Gdf5-Cre/Bmpr1a^floxP^* mutant embryos showed obvious retention of interdigital webbing between the first and second, but not other, digits of E14.5 forelimbs (Figure 2A and 2B), a pattern that corresponds to the presence or absence of webbing seen in the adult limb. They also showed excess tissue on the posterior margin of the fifth digit (Figure 2B, arrow). Analysis of LACZ expression in *Gdf5-Cre/R26R* reporter embryos showed that Cre-mediated recombination has occurred by E13.5 in the metacarpal-phalangeal joints, and in the interdigital region between the first and second, but not other, digits. In addition, a domain of recombination and expression of LACZ is also reproducibly seen in the posterior half of the fifth digit (Figure 2C). Terminal deoxynucleotidyl transferase–mediated deoxyuridine triphosphate nick end labeling (TUNEL) staining of interdigital mesenchyme between the first and second digits (Figure 2D and 2E) and the fifth digit flanking mesenchyme showed a decreased number of dying cells in the regions where excess tissue is retained in the mutant limbs. Numbers of phosphorylated histone H3-labeled proliferating cells were also elevated in these regions (Figure 2F). Most cells found in the webbed region between the first and second digits at E15.5 strongly expressed LACZ in *Gdf5-Cre/Bmpr1a^floxP^* mutant embryos (Figure 2H). These data suggest that regional loss of BMPR1A receptor signaling blocks programmed cell death in interdigital mesenchyme, and that the recombined cells survive and proliferate in the absence of BMPR1A signaling.

### Failure of Early Joint Formation in Ankle Regions

**Figure 4:**
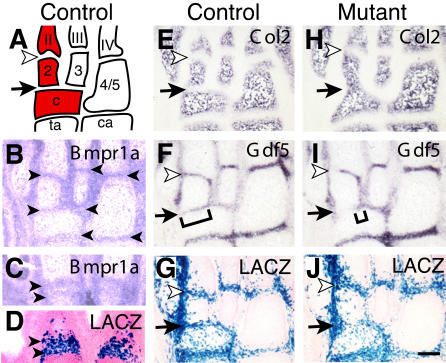
*Bmpr1a* Is Expressed in Joints and Is Required for Continued Joint Formation in the Ankle Region (A) Diagram of ankle bones from a wild-type mouse; bones fusing in mutant are colored red. Roman numerals II–IV, metatarsals; 2, 3, and 4/5, distal row of tarsal bones; c, central tarsal bone; ta, talus; ca, calcaneus. (B and C) In situ hybridization at E15.5 showing that *Bmpr1a* is expressed in ankle joint interzones (B, arrowheads) and in the forming articular regions of the phalangeal joints (C, arrowheads). (D) Near adjacent section to (C) showing *Gdf5-Cre* induced LACZ expression from *R26R* in the forming joints of the digits (arrowheads). (E–J) Marker gene expression and *R26R* LACZ staining patterns on near adjacent sections of control and mutant embryos. In control mice at E15.5 ankle joints are clearly delineated as regions that have down-regulated *Col2* (E), express *Gdf5* throughout (F), and express LACZ in most cells (G; white arrowheads and black arrows). In mutant embryos at the same stage, joint formation is incomplete. Faint *Col2* expression can be seen connecting a medial region of tarsal 2 with metatarsal II (H, white arrowhead), and *Gdf5* expression does not extend all the way across the joint at this location (I, white arrowhead). Between tarsals c and 2, mutants express *Col2* across the normal joint-forming region (H, black arrow) and lack expression of *Gdf5* at sites where skeletal fusions are observed (I, black arrow and bracket). (J) Scale bar = 100 μm.

The *Bmpr1a* gene is expressed in the interzone region of developing joints at E13.5 (Baur et al. 2000). In situ hybridization showed that the gene is also expressed in the interzones of ankle joints and prospective articular cartilage regions of digit joints at E15.5 (Figure 4). LACZ staining indicated that Cre-mediated recombination begins to occur in ankle joints around E14.5, and is extensive by E15.5 (Figure 4G and 4J) (unpublished data). In the ankle joint regions that were obviously fused in postnatal mutant animals, alterations in early joint marker expression could also be seen by E15.5. At this stage, the *Gdf5* gene is normally expressed in stripes that mark the sites of joint formation (Figure 4F), and the gene for the major collagen protein of cartilage matrix *(Col2a1)* is down-regulated in the interzone region (Figure 4E). In contrast, *Col2a1* staining extended completely through the joint region between the second and central tarsal of *Gdf5-Cre/Bmpr1a^floxP^* mutants (Figure 4H, black arrow), and *Gdf5* expression was seen only as a small notch extending into where the joint should be forming (Figure 4I, bracket). These data suggest that the fusions seen between ankle bones in postnatal mutant skeletons are the result of incomplete segmentation of skeletal precursors during embryonic development, a defect confined to some locations in the ankle.

### Failure to Maintain Articular Cartilage in Other Joints

In most joints of *Bmpr1a* conditional knockout mice, embryonic segmentation of skeletal precursors occurred normally. Although *Gdf5-Cre*-mediated recombination was seen as early as E13.5 in digit interzone regions (see Figure 2C), no changes in cell death or cell proliferation could be seen in the metacarpal-phalangeal or metatarsal-phalangeal joints at E13.5 or E14.5 (unpublished data). Similarly, although clear LACZ expression was seen by E15.5 in interphalangeal joints and periarticular regions (Figure 4D), no difference in morphology or expression of *Col2a1, Gdf5,* or *Bmpr1b* was seen in the articular regions of the phalanges at these stages (unpublished data).

**Figure 5:**
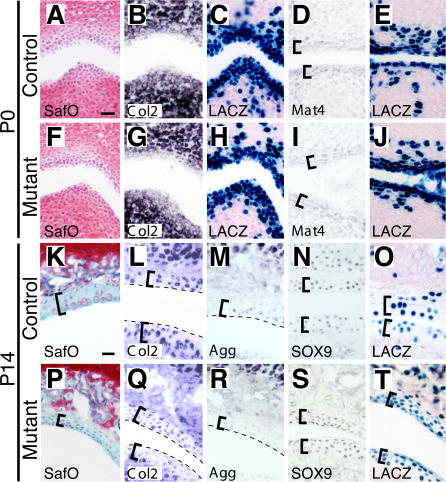
*Bmpr1a* Is Required to Maintain Expression of ECM Components in Articular Cartilage In situ hybridization or LACZ staining on near adjacent sections of metacarpal-phalangeal joints (A–C and F–H) and the tarsal 2-metatarsal II joint (D–E and I–J) of P0 mice. At birth, articular cartilage of controls (A–E) and mutants (F–J) appears similar by Safranin O staining (A and F), and *Col2* expression (B, G). *Mat4* expression confirms that articular cartilage is initially specified in mutants (D andI, brackets). LACZ expression confirms Cre-mediated recombination has occurred in articular cartilage (C, H, E, and J). (K–T) Near adjacent sections of the metacarpal-phalangeal joints of P14 mice. Two weeks after birth, articular cartilage of controls stains with pericellular Safranin O (orange staining, K), and expresses *Col2* (L), *Agg* (M), and SOX9 (N). In contrast, mutant articular cells are smaller and more densely packed, lack pericellular Safranin O staining (P), have reduced expression of *Col2* (Q) and *Agg* (R), but retain normal levels of SOX9 protein (S, brackets; dashed line marks faint edges of articular surfaces). LACZ expression confirms Cre-mediated recombination has occurred in articular cells (O ansd T, brackets). (A and K) Scale bar = 75 μm.

At birth, digit joints were generally indistinguishable from those in control animals; chondrocytes were abundant in articular regions and were surrounded by typical cartilage matrix with normal staining by Safranin O, a histological stain for proteoglycans (Figure 5). At this stage, both wild-type and mutant cells in articular regions also expressed high levels of *Col2a1* and *Aggrecan (Agg),* the genes encoding the major structural proteins of cartilage matrix (Figure 5B and 5G) (unpublished data). No alterations in cellular apoptosis or proliferation were observed (unpublished data).

To determine whether articular cells were properly specified in mutants, we also analyzed expression of *Matrilin-4 (Mat4),* a gene expressed specifically in the periarticular and perichondral regions of developing joints (Klatt et al. 2001). In both control and mutant animals, transcription of *Mat4* was clearly detectable in the articular cartilage layers of newborn joints (Figure 5D and 5I). In all experiments, expression of LACZ throughout articular regions indicated that Cre-mediated recombination had occurred throughout the articular regions (Figure 5C, 5H, 5E, and 5J). The normal histological appearance, staining properties, and marker gene expression patterns suggest that *Bmpr1a* is not required for the initial formation or specification of articular cartilage.

By 1 wk after birth, obvious differences began to be detected in the articular regions of mutant animals. The expression of *Col2a1* was reduced throughout the articular surfaces of the carpals, metacarpals, and phalanges of the forefeet (unpublished data). Less severe reductions were also seen in articular cells of tarsals and metatarsals in the hindfeet (unpublished data). By 2 wk of age, *Col2a1* expression was reduced in most cells of the articular region (Figure 5L and 5Q), accompanied by markedly reduced Safranin O staining (Figure 5K and 5P), and decreased expression of *Agg* and two genes normally expressed in more mature articular cartilage cells, *Collagen 3 (Col3a1)* and *Collagen 10 (Col10a1)* (Figure 5M and 5R) (unpublished data) (Eyre 2002). Inhibition of BMP signaling in cultured chondrocytes has previously been reported to induce *Collagen 1 (Col1a1)* expression, increase proliferation, and result in cells with flattened, fibroblast-like morphology (Enomoto-Iwamoto et al. 1998). However, we saw no increase in the expression of *Col1a1* in mutant articular cartilage, and no proliferation was detected in articular cells of either mutant or control animals (unpublished data). While recombined LACZ marker expression was detected in most articular cartilage cells, it was also observed in scattered subarticular chondrocytes, growth plate chondrocytes, and osteoblasts (Figure 5O and 5T) (unpublished data). Although this implies that BMP signaling was defective in multiple cell types, the observed defects were confined to the articular cartilage. For example, *Osteocalcin* and *Col1a1* expression appeared normal in osteoblasts (unpublished data). Together, these data suggest that BMPR1A activity is required in postnatal joint articular cartilage to maintain expression of many genes encoding structural components of cartilage matrix.

Previous studies have shown that *Sox9* is required for normal cartilage differentiation, for expression of cartilage extracellular matrix (ECM) genes including *Agg*, and is a direct transcriptional regulator of the key cartilage matrix gene *Col2a1* (Bell et al. 1997; Lefebvre et al. 1997; Bi et al. 1999; Sekiya et al. 2000). Notably, despite reduced expression of many cartilage matrix marker genes in *Bmpr1a* mutant mice, the SOX9 protein was present at normal levels in articular regions at all stages examined, including newborn, 2-wk-old, 7-wk-old, and 9-mo-old mice (Figure 5N and 5S) (unpublished data).

### Synovial Hypertrophy, Cartilage Erosion, and Accelerated Cartilage Maturation

**Figure 6:**
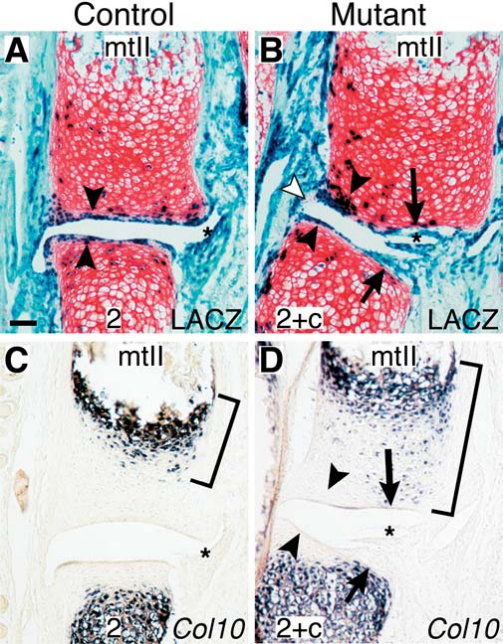
Synovial Membrane Expansion, Articular Surface Erosion, and Accelerated Maturation of Underlying Cartilage in Ankles of *Bmpr1a* Mutant Mice Near adjacent sections from the tarsal 2-metatarsal II joint of 7-d-old mice. (A and B) LACZ staining (blue) shows Cre-mediated recombination is largely restricted to articular (arrowheads) and synovial cells (asterisks) in both controls and mutants. (C and D) In situ hybridization shows *Col10* expression expands in mutants toward regions of synovial membrane expansion and articular surface erosion (brackets and arrows). This may be a cell nonautonomous effect of joint damage, since the LACZ expressing cells at the articular surface do not show upregulation of *Col10* (arrowheads) and the region of expanded *Col10* expression is largely made up of cells that have not undergone Cre-mediated recombination. Note the formation of a cartilaginous bridge along the joint capsule of the mutant where joint formation is disrupted at earlier stages (B, white arrowhead, and Figure 3, white arrowheads). (A) Scale bar = 75 μm.

Conditional loss of *Bmpr1a* led to marked hypertrophy of the synovial membrane in the joint capsule of some joints, particularly in the ankle region. In the most severely affected joints, the expanded synovial membrane grew into the joint space and was associated with obvious loss or erosion of the articular cartilage (Figure 6A and 6B, asterisks, arrows). Accelerated cartilage maturation and increased expression of *Col10a1* was frequently seen in the chondrocytes underlying the articular erosions (Figure 6C and 6D, brackets) (unpublished data). Interestingly, the regions of increased *Col10a1* expression did not correspond to the regions that had undergone Cre-mediated recombination. Instead, increased expression of *Col10a1* was seen in a zone of largely LACZ-negative cells stretching from the cartilage adjacent to the ossification front (where *Col10a1* is normally expressed in maturing cartilage cells), toward the regions where surface articular cartilage was severely eroded or missing (Figure 6A and 6B, arrowheads). Previous studies suggest that parathyroid hormone-related protein, a diffusible signal made in the articular surface, may normally inhibit maturation of underlying cartilage (Vortkamp et al. 1996; Weir et al. 1996). Local loss of the articular surface could remove this inhibition and lead to a cell-nonautonomous acceleration of maturation in chondrocytes underlying points of articular erosion.

This synovial hypertrophy is associated with increased numbers of mononuclear cells resembling synoviocytes or macrophages, cell types that are difficult to distinguish even with surface markers at early postnatal stages. However, no neutrophils were observed, suggesting that there is little inflammation. At later stages synovial hypertrophy is reduced. Further work will be needed to determine whether synovial development is regulated by BMP signaling, or whether the synovium becomes enlarged as a response to nearby skeletal malformations (such as fusion of the second and central tarsals or defects in the articular cartilage).

### Noninflammatory Degeneration of Articular Cartilage in Digit and Knee Joints

**Figure 7:**
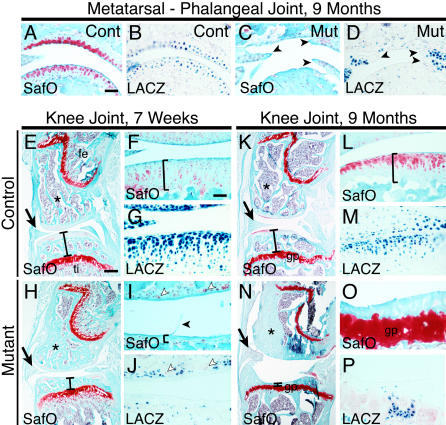
Loss of *Bmpr1a* Signaling Leads to Articular Cartilage Fibrillation and Degeneration in Digits and Knees of Aging Mice (A–D) Near adjacent sections of metatarsal-phalangeal joints from 9 month old mice. Articular cartilage of controls is complete and stains strongly with Safranin O (A, orange stain). In contrast, articular cells of mutants are severely fibrillated or absent with much reduced staining of Safranin O (C, arrowheads). LACZ expression confirms Cre-mediated recombination has occurred in articular cells (B and D). (E–P) Sagittal sections through knee joints of 7-wk- (E–J) or 9-mo-old animals (K–P); fe, femur; ti, tibia; gp, growth plate. Seven weeks after birth, the height of the tibial epiphysis is reduced in mutants (E and H, bars), and their articular layer stains poorly with Safranin O, is fibrillated, and is strikingly thinner (F and I, black arrowhead, and brackets). Near adjacent sections with LACZ staining confirm Cre-mediated recombination has occurred in articular cells (G and J). Note that in mutants, LACZ is absent in cells adjacent to those that do stain with Safranin O, suggesting *Bmpr1a* may act cell autonomously (I and J, white arrowheads). At 9 mo old, the mutant tibial epiphysis is extremely thin (K and N, bars), and the articular layer is completely absent, leaving bone to rub directly on bone (L and O, bracket). LACZ staining shows Cre-mediated recombination occurred in articular cells of controls (M) and in some remaining skeletal tissue of mutants (P). Also note aberrantly formed meniscal cartilage in mutants (E, H, K, and N, arrows), and increased sclerosis in mutant epiphyses (E, H, K, and N, asterisks). (A and K) Scale bar = 50 μm; (I) scale bar = 300 μm.

Outside of the ankle region, little or no evidence was seen for expansion of the synovial membrane. Instead, mutant mice showed histological signs of osteoarthritis, such as fibrillation of the articular surface (Figure 7). As previously seen in 1- and 2-wk-old animals, Safranin O staining and *Agg* and *Col10* expression were all reduced in mutant articular regions of the forefeet and hindfeet by 7 wk of age, and the beginning signs of cartilage loss were observed (unpublished data). By 9 mo of age, many regions of articular cartilage were completely missing or extremely fibrillated, leaving regions of exposed bone on the surface (Figure 7A–7D). No alterations were seen in the expression of *Osteocalcin, Col1a1,* or matrix metalloprotease-13 at either 7 wk or 9 mo.

The major weight-bearing joint of the hindlimb, the knee, showed changes that closely paralleled that seen in the foot joints. All markers of cartilage matrix looked similar to controls at E16.5, suggesting that early stages of joint formation were not disrupted (unpublished data). By postnatal day 7, Safranin O staining and *Col2a1* and *Agg* expression were clearly reduced in the mutant, despite continued expression of Sox9 (unpublished data). The overall shape of mutant knee skeletal elements appeared similar to controls, although the fibrocartilaginous meniscus that resides between the femur and tibia appeared much less dense in mutants at E16.5. Some cartilage formed in the meniscus region, but the size of these elements was greatly reduced and contained abundant cells with fibrous, noncartilaginous appearance (unpublished data). This reduction of the meniscus can also be seen in sections from 7-wk- and 9-mo-old animals (Figure 7E, 7H, 7K, and 7N, arrows).

At 7 wk of age the normally domed tibial epiphysis was flattened and depressed in the knees of mutant animals, markedly reducing the distance between the growth plate and articular surface (Figure 7E and 7H, vertical bar). Articular cartilage was also thinner than in control animals, showed nearly complete absence of Safranin O staining, and was either acellular or beginning to fibrillate in many regions (Figure 7F and 7I). The few large Safranin O-stained cells still apparent in mutant articular regions appeared to correspond in position to rare LACZ-negative cells in adjacent sections, suggesting that *Bmpr1a* is required cell-autonomously in articular cartilage (Figure 7I and 7J, white arrowheads). By 9 mo, large areas of mutant knees were devoid of articular cells, and the bones of the femur and tibia appeared to rub directly against each other. Furthermore, the epiphysis of the tibia was extremely depressed, to the point that growth plate cartilage was almost exposed through the surface of the bone (Figure 7K, 7L, 7N, and 7O). In addition, mutants at 7 wk and 9 mo showed subchondral sclerosis, especially in the epiphysis of the femur (Figure 7E, 7H, 7K, and 7N, asterisks). While subchondral sclerosis is commonly seen in cases of osteoarthritis, it is unclear in this case whether the sclerosis is mainly a response of bone formation to compensate for decreased articular cartilage, or whether it is the effect of loss of *Bmpr1a* signaling in some LACZ-positive cells that are also observed in these regions (unpublished data).

The histological signs of joint arthritis were accompanied by functional impairments in both grasping ability and range of motion in mutant animals. *Gdf5-Cre/Bmpr1a^floxP^* mutant animals showed a highly significantly reduced ability to grasp and remain suspended on a slender rod (mean suspension time: controls 38 ± 6 s, *n* = 39; mutants 6 ± 3 s, *n* = 11; *p* < 0.0001). Mutant mice also showed a clear decrease in the maximum range of mobility of two different joints in the digits, as assayed by passive manipulation (MT/P1 joint: controls 100 ± 0°, *n* = 26; mutants 82 ± 3°, *n* = 8; *p* < 0.0003; P1/P2 joint: controls 152 ± 1°, *n* = 23; mutants 140 ± 5°, *n* = 6; *p* < 0.05). The structural, histological, marker gene expression, and functional changes in mutant mice demonstrate that BMPR1A is required for normal postnatal maintenance of articular cartilage.

## Discussion

Previous studies suggest that BMP signaling is involved in a large number of developmental events. Many of these events occur early in embryogenesis, and complete inactivation of BMP receptors causes death by E9.5 (Mishina et al. 1995). The *Gdf5-Cre* recombination system bypasses the early embryonic lethality of *Bmpr1a* mutations, and provides important new information about the role of this receptor in limb and skeletal development.

The three major limb phenotypes revealed by eliminating *Bmpr1a* with *Gdf5*-driven Cre include webbing between digits, lack of joint formation at specific locations in the ankle, and failure to maintain articular cartilage after birth, resulting in severe arthritis. Previous studies have shown that manipulation of BMP signaling alters interdigital apoptosis during development of the limb, but no experiment has identified a specific member of the BMP signaling pathway that is required for this process (Yokouchi et al. 1996; Zou and Niswander 1996; Zou et al. 1997; Guha et al. 2002). Our new loss-of-function data confirm that BMP signaling is required for interdigital apoptosis and suggests that *Bmpr1a* is a critical component for mediating this signal.

At some sites, loss of *Bmpr1a* function leads to a defect in the early stages of joint formation, resulting in a complete failure to form a joint and fusion of bones in the ankle. Mutations in two different ligands in the BMP family, *Gdf5* and *Gdf6,* the *Bmpr1b* receptor, and in the human *Noggin* locus (Storm and Kingsley 1996; Gong et al. 1999; Baur et al. 2000; Yi et al. 2000; Settle et al. 2003) also produce defects in joint formation at specific locations in the limbs. The joint defects associated with multiple components of the BMP pathway provide strong evidence that BMP signaling is required for early stages of joint formation at some anatomical locations.

Most joints still form normally when *Bmpr1a* is knocked out in *Gdf5* expression domains. The lack of joint fusions outside the ankle region could be due to differences in requirement for BMP signaling in different joints, to compensating expression of other BMP receptors outside the ankles, or to differences in the detailed timing of *Gdf5-Cre* stimulated gene inactivation in ankles and other joint regions. Comparison of the expression of the HPLAP marker (driven directly by *Gdf5* control elements) and the *R26R* LACZ marker (expressed following *Gdf5-Cre* recombination) suggests that recombination-stimulated changes in gene expression may be delayed for a 0.5–1 d in the digit region (see Figure 1C). In addition, levels of *Bmpr1a* mRNA and protein may persist for some time following *Gdf5-Cre* stimulated recombination, making it possible to bypass an early requirement for *Bmpr1a* in joint formation at some locations.

Following the decay of *Bmpr1a* mRNA and protein, the *Gdf5-Cre* strategy should result in permanent inactivation of *Bmpr1a* function in recombined cells. This system thus provides one of the first strong genetic tests of *Bmpr1a* function at later stages of joint development. Despite the normal appearance of articular regions and gene expression immediately after birth, *Bmpr1a*-deficient animals are unable to maintain the normal differentiated state of articular cartilage as they continue to develop and age. These results suggest that BMP receptor signaling is essential for continued health and integrity of articular cartilage in the postnatal period.

Articular cartilage is a key component of synovial joints and is one of the few regions in the skeleton where cartilage is maintained into adulthood. Despite the importance of articular cartilage in joint health and mobility, little is known about the factors that create and maintain it in thin layers at the ends of long bones. In our experiments, articular cartilage lacking *Bmpr1a* retains some normal characteristics, in that it maintains a very low proliferation rate, does not express *Col1a1,* and continues to express SOX9, a major transcription factor regulating expression of structural components of cartilage matrix. However, several of the most prominent structural components of cartilage matrix fail to be maintained in mutant animals, resulting in decreased synthesis of *Col2a1, Agg,* and proteoglycans. Therefore, BMPR1A appears to maintain articular cartilage primarily through inducing expression of key ECM components.

It is interesting that the SOX9 transcription factor continues to be expressed in mutant cartilage despite loss of *Col2a1,* a direct target of this transcription factor (Bell et al. 1997; Lefebvre et al. 1997). Previous studies suggest that SOX9 activity can be modified by protein kinase A (PKA)-dependent protein phosphorylation, or by coexpression of two related proteins, L-SOX5 and SOX6 (Lefebvre et al. 1998; Huang et al. 2000). In addition, close examination of the order of genes induced during chicken digit formation reveals that *Sox9* turns on first, followed by *Bmpr1b* with *L-Sox5,* and then *Sox6* and the cartilage matrix structural components *Col2a1* and *Agg* (Chimal-Monroy et al. 2003). These results, together with the altered pattern of gene expression seen in our *Bmpr1a*-deficient mice, suggest that BMPR1A signaling may normally act to stimulate SOX9 by post-translational protein modification, or to induce *L-Sox5* or *Sox6* in cartilage to maintain expression of ECM components. These models are consistent with the ability of BMP2 to both increase PKA activity and induce expression of *Sox6* in tissue culture cells (Lee and Chuong 1997; Fernandez-Lloris et al. 2003). Although we have tried to monitor the expression of *L-Sox5* or *Sox6* in postnatal articular cartilage, and test the phosphorylation state of SOX9 using previously described reagents (Lefebvre et al. 1998; Huang et al. 2000), we have been unable to obtain specific signal at the late postnatal stages required (unpublished data). Furthermore, null mutations in *L-Sox5* or *Sox-6* cause lethality at or soon after birth, and no effect on cartilage maintenance has been reported (Smits et al. 2001). However, it seems likely that these or other processes regulated by BMP signaling cooperate with SOX9 to induce target genes in articular cartilage.

Mutation of *Smad3* or expression of dominant negative transforming growth factor β (TGF-β) type II receptor also disrupts normal articular cartilage maintenance (Serra et al. 1997; Yang et al. 2001). Both manipulations should disrupt TGFβ rather than BMP signaling, and both manipulations cause articular cartilage to hypertrophy and be replaced by bone. In contrast, our analysis of *Bmpr1a* mutant articular cartilage showed a loss of ECM components, but no signs of hypertrophy or bone replacement. Therefore, TGFβ and BMP signaling are playing distinct but necessary roles to maintain articular cartilage.

Although BMPs were originally isolated on the basis of their ability to induce ectopic bone formation, their presence in articular cartilage and strong effect on cartilage formation has stimulated interest in using them to repair or regenerate cartilage defects in adult animals (Chang et al. 1994; Erlacher et al. 1998; Edwards and Francis-West 2001; Chubinskaya and Kuettner 2003). The failure to maintain articular cartilage in the absence of normal BMPR1A function suggests that ligands or small molecule agonists that interact specifically with this receptor subtype may be particularly good candidates for designing new approaches to maintain or heal articular cartilage at postnatal stages.

Lack of *Bmpr1a* function in articular cartilage results in severe fibrillation of the articular surface and loss of joint mobility. The development of severe arthritis symptoms in *Bmpr1a*-deficient mice raises the possibility that defects in BMP signaling also contribute to human joint disease. Osteoarthritis is known to have a significant genetic component, but it likely involves multiple genetic factors that have been difficult to identify (Spector et al. 1996; Felson et al. 1998; Hirsch et al. 1998). Humans that are heterozygous for loss-of-function mutations in *BMPR1A* are known to be at risk for juvenile polyposis (Howe et al. 2001; Zhou et al. 2001), but the risk of osteoarthritis for these people has not been reported. However, the control mice used in this study were heterozygous for a null allele of *Bmpr1a,* and they showed little sign of osteoarthritis even late in life. Several chromosome regions have been previously linked to arthritis phenotypes in humans using either association studies in populations or linkage studies in families. It is interesting to note that several of these chromosome regions contain genes encoding different members of the BMP signaling pathway, including the *BMP5* gene on human chromosome 6p12 (Loughlin et al. 2002), the *MADH1* gene on human chromosome 4q26–4q31 (Leppavuori et al. 1999; Kent et al. 2002), and the *BMPR2* receptor on human chromosome 2q33 (Wright et al. 1996). The complex nature of human osteoarthritis suggests that interactions between multiple genes may be involved in modifying susceptibility to the disease. The inclusion of genetic markers near BMP signaling components may help identify additional osteoarthritis susceptibility loci and facilitate the search for causative mutations.

Development and disease processes in synovial joints have been difficult to study genetically, because synovial joints are generated and function at relatively late stages of vertebrate development. The *Gdf5-Cre* system provides a new method for restricting gene expression or inactivation primarily to articular regions, thus avoiding the pleiotropic functions of many genes in other tissues. Depending on the configuration of the floxed target gene, this system can be used to either activate the expression of a gene primarily in developing joints (ssee Figure 1B–1D), or to inactivate gene function in articular regions (see Figure 3). Additional studies with this system should greatly enhance our knowledge of the development, function, and disease mechanisms of joints, and may bring us closer to better prevention and treatment of joint diseases.

## Materials and Methods

### 

#### Generation of *Gdf5-Cre* transgenic mice

A mouse 129x1/SvJ BAC library (Invitrogen) was screened to identify a 140-kb BAC from the *Gdf5* locus. This BAC was modified using a homologous recombination system in E. coli (Yang et al. 1997) to place nuclear-localized Cre recombinase (from plasmid pML78, gift of Gail Martin) followed by IRES-*hPLAP* (from plasmid 1726, gift of Oliver Bogler) directly behind the ATG start site of *Gdf5*. In the process, 583 bp of the first exon of *Gdf5* was removed and no functional GDF5 protein is predicted to be produced. The 5′ homology arm was subcloned from a PCR product tailed with XhoI and Bsp120I restriction sites that contains 781 bp of 5′ genomic *Gdf5* sequence ending at the ATG translation start site (forward primer 5′-CTGTCTCGAGATGAGGTGGAGGTGAAGACCCC-3′; reverse 5′-GTTTGGGCCCATCCTCTGGCCAGCCGCTG-3′). *Cre* was subcloned from a 1.1-kb Bsp120I/EcoRI fragment of pML78. IRES *hPLAP* was subcloned from a 2.1-kb PCR product tailed with EcoRI and SpeI sites that contains the *hPLAP* translation stop site (forward primer 5′-ATCTCTCGAGGAATTCTCCACCATATTGCCGTCTTTTG-3′; reverse 5′-AGAACTCGAGACTAGTCGGGACACTCAGGGAGTAGTGG-3′). The 3′ homology arm was subcloned from a 0.8-kb PCR product amplified from a 0.9-kb XhoI *Gdf5* genomic subclone containing part of the first exon and downstream intron. The forward primer contains the 3′ end of the first exon and is tailed with a SpeI site; the reverse primer is from the T7 promoter of the vector containing the 0.9-kb subclone and flanks the intronic XhoI site (forward primer 5′-CTAAACTAGTCACCAGCTTTATTGACAAAGG-3′; reverse 5′-GATTTCTAGAGTAATACGACTCACTATAGGGC-3′). The targeting construct was built and verified in pBSSK (Stratagene, La Jolla, California, United States), then digested with XhoI and subcloned into pSV1, the vector used for homologous recombination (Yang et al. 1997). Southern blotting, PCR, and DNA sequence analysis confirmed the appropriate targeting construct and BAC modifications were made (unpublished data).

Before the modified BAC was injected to produce transgenic animals, a *loxP* site present in the BAC vector, pBeloBAC11, was removed to prevent the addition of undesired Cre target sites into the genome. To do this, BAC DNA was prepared by CsCl separation, digested with NotI to free the insert from the vector, and size-fractionated over a sucrose gradient. Aliquots of fractions were run on a pulse-field gel and Southern blotted using vector-specific DNA as a probe. Fractions containing unsheared insert and almost no detectable vector DNA were dialyzed in microinjection buffer (10 mM Tris [pH 7.4] with 0.15 mM EDTA [pH 8.0]) using Centriprep-30 concentrators (Millipore, Billerica, Massachusetts, United States). This purified insert DNA was adjusted to 1 ng/μl and injected into the pronucleus of fertilized eggs from FVB/N mice by the Stanford Transgenic Facility. Transgenic founder mice were identified by PCR using *Cre*-specific primers 5′-GCCTGCATTACCGGTCGATGCAACGA-3′ and 5′-GTGGCAGATGGCGCGGCAACACCATT-3′, which amplify a 725-bp product, and were assessed for absence of BAC vector using vector-specific primers 5′-CGGAGTCTGATGCGGTTGCGATG-3′ and 5′-AGTGCTGTTCCCTGGTGCTTCCTC-3′, which amplify a 465-bp product. Three lines of *Gdf5-Cre* mice were established and maintained on the FVB background. Matings with R26R Cre-inducible LACZ reporter mice (Soriano 1999) were used to test for Cre activity.

Staining for LACZ and HPLAP on whole embryos or sections of embryos was accomplished following established protocols (Lobe et al. 1999). The red LACZ substrate (see Figure 1E) is 6-chloro-3-indoxyl-beta-D-galactopyranoside (Biosynth International, Naperville, Illinois, United States).

#### General characterization of *Bmpr1a* mutant mice

*Bmpr1a* null and floxed alleles (Ahn et al. 2001; Mishina et al. 2002) were obtained on a mixed 129 and C57BL/6 background and maintained by random breeding. Mice carrying the null and floxed alleles were typically mated to *Gdf5-Cre* mice as shown in Figure 3. The resulting mice are on a mixed 129; C57Bl/6; FVB/N background, with both controls and mutant animals generated as littermates from the same matings. Whole-mount skeletal preparations were made from 34- to 36-d-old mice (Lufkin et al. 1992). Pairs of ears from euthanized 6-mo-old animals were removed, pinned, photographed, projected, and measured from the base of the curve formed between the tragus and antitragus to the farthest point at the edge of the pinnae. Grasping ability in 6-mo-old mice was measured by placing animals on a slender rod and timing how long they could remain suspended on the rod, to a maximum time allowed of 2 min. Data from five consecutive trials for each mouse were averaged. Range of motion assays were conducted on the MT/P1 and P1/P2 joints of the second hindlimb digit from euthanized 18-wk-old animals. Forceps were used to bend the joint to its natural stopping position, and the resulting angle was measured to the nearest 10° under 12.5× magnification using a 360° reticule. Analysis described in this section occurred on animals lacking *R26R*. Control mice included all nonmutant genotypes generated by Parent 1 being heterozygous for *Gdf5-Cre* and *Bmpr1a^null^* and Parent 2 being heterozygous for *Bmpr1a^floxP^* (see Figure 3). All statistical analysis used the Student's t-test or Welch's t-test, and values listed are mean ± standard error of the mean.

#### Cell death and proliferation assays

Limbs from mutant and control animals at E13.5 and E14.5 were dissected and frozen in OCT (Sakura Finetek,Torrence, CA, United States). Cryosections of tissue were assayed by TUNEL using the In Situ Cell Death Detection Kit, Fluorescein (Roche, Basel, Switzerland). Following TUNEL, slides were washed in PBS, blocked with PBS + 0.05% Tween-20 + 5% goat serum, washed again, and incubated with a 1:200 dilution of a rabbit anti-phospho-histone-H3 antibody called Mitosis Marker (Upstate Biotechnology, Lake Placid, New York, United States) to identify cells in mitosis. Cy3-labeled anti-rabbit secondary antibody was used to detect the antibody. Cell nuclei were labeled with DAPI, and slides were mounted in Vectamount (Vector Laboratories, Burlingame, California, United States) and visualized at 100× magnification. The area of selected anatomical sites were measured, and the number of TUNEL-labeled nuclear fragments and the number of Cy3-labeled nuclei were counted from three 10-μm sections spanning 50 μm, from three control and three mutant animals. The number of labeled cells in the metacarpal-phalangeal and metatarsal-phalangeal joints was counted in a 290 μm × 365 μm rectangle placed around the center of the joint. The posterior region of the fifth digit was defined by drawing a line from the tip of the digit down 2.15 mm and across to the lateral edge of the tissue. For this analysis, the *R26R* Cre reporter was not present.

#### Histology and histochemistry

Tissue from animals ranging from stages E14.5 to P14 was prepared for analysis by fixing in 4% paraformaldehyde (PFA) in PBS for 45 min to 4 h depending on the stage; washing three times in PBS, once in PBS + 15% sucrose for 1 h, and once in PBS + 30% sucrose for 2 h to overnight depending on the stage; and then freezing in OCT. Tissue from animals aged 7 wk to 9 mo was processed similarly to earlier stages except that it was decalcified in 0.5 M EDTA (pH 7.4) for 4 d prior to incubating in sucrose. All solutions were prechilled and used at 4 °C with agitation, and skin from tissues of P0 or older mice was lacerated or removed prior to processing.

Tissue was then cryosectioned at 12 μm and processed. Staining of sections with Safranin O, Fast Green, and Harris' hematoxylin was carried out using standard histological procedures. Detection of LACZ activity with X-Gal was performed as described (Lobe et al. 1999) and was followed by refixing in 4% PFA, rinsing with deionized water, counterstaining with Nuclear Fast Red (Vector Labs), rinsing with water again, and then mounting in Aquamount (Lerner Labs, Pittsburgh, Pennsylvania, United States).

RNA in situ hybridization was performed as described (Storm and Kingsley 1996), with the following modifications: (1) Prior to the acetylation step, sections were incubated with 10–20 μg/ml proteinase K for 30 s to 7 min at room temperature (depending on the developmental stage), followed by refixing in 4% PFA and washing three times in PBS; (2) prehybridization step was skipped, and (3) embryonic tissue sections used a different color development mix (Thut et al. 2001). Probes for the following genes have been published previously: *Bmpr1a* (Mishina et al. 1995), *Col2a1* (Metsaranta et al. 1991), *Col10a1* (Apte et al. 1992), *Gdf5* (Storm and Kingsley 1996), *Osteocalcin* (Celeste et al. 1986), and *Sox5* and *Sox6* (Lefebvre et al. 1998). The following probe templates were gifts: *Agg*, Dr. Vicki Rosen, Genetics Institute; *Bmp2* and *Bmp4*, Arend Sidow, Stanford University; *Col1a1*, Bjorn Olsen, Harvard Medical School; *Bmpr1b, Col3a1,* and *Mat4* probes were made from ESTs with IMAGE clone numbers 5056341, 478480, and 406027, respectively (Invitrogen, Carlsbad, California, United States).

Sections for immunohistochemistry were fixed in 4% PFA, then digested with 942–2,000 U/ml type IV-S bovine hyaluronindase (Sigma, St. Louis, Missouri, United States) in PBS (pH 5) at 37 °C for 30 min to 2 h depending on the stage. Slides were then washed in PBS, treated with 0.3% hydrogen peroxide in 100% methanol for 30 min, washed, blocked with PBS + 0.05% Tween20 + 5% goat or fetal bovine serum, washed again, and incubated with primary antibodies in PBS + 0.05% Tween 20 + 1% goat or fetal bovine serum overnight at 4 °C. Biotin-labeled secondary antibodies (Vector Labs) were tagged with HRP using the Vectastain Elite ABC kit (Vector Labs) followed by detection with DAB (Vector Labs). Primary antibodies and dilutions used were: goat anti-mouse MMP13, 1:100 (Chemicon International, Temecula, California, United States); rabbit anti-human SOX9, 1:500 (Morais da Silva et al. 1996); rabbit anti-phosphorylated-SOX9 (SOX9.P), 1:10–1:250 (Huang et al. 2000).

## Supporting Information

### Accession Numbers

GenBank (http://www.ncbi.nih.gov/Genbank/) accession numbers for the genes discussed in this paper are *Gdf5* (AC084323) and *Bmpr1a* (NM_009758).

## Abbreviations

BAC: bacterial artificial chromosome
Bmpr1a: bone morphogenetic protein receptor 1a
E[number]: embyonic day [number]
ECM: extracellular matrix
GAC: transgenic line carrying Gdf5-alkaline phosphatase-Cre construct
Gdf5: growth differentiation factor 5
hPLAP: human placental alkaline phosphatase
IRES: internal ribosome entry site
PFA: paraformaldehyde
R26R: lacZ ROSA26 Cre reporter strain
TGF-β: transforming growth factor β
TUNEL: terminal deoxynucleotidyl transferase–mediated deoxyuridine triphosphate nick end labeling
